# In Vivo Safety of New Coating for Biodegradable Magnesium Implants

**DOI:** 10.3390/ma16175807

**Published:** 2023-08-24

**Authors:** Bohdan Dryhval, Yevheniia Husak, Oksana Sulaieva, Volodymyr Deineka, Mykola Pernakov, Mykola Lyndin, Anatolii Romaniuk, Wojciech Simka, Maksym Pogorielov

**Affiliations:** 1 Biomedical Research Centre, Sumy State University, R-Korsakova Street, 40007 Sumy, Ukraine; drigval007@gmail.com (B.D.); yevheniia.husak@polsl.pl (Y.H.); v.deineka@med.sumdu.edu.ua (V.D.); m.pernakov@med.sumdu.edu.ua (M.P.); n.lyndin@med.sumdu.edu.ua (M.L.); a.romanyuk@med.sumdu.edu.ua (A.R.); 2 Faculty of Chemistry, Silesian University of Technology, 44-100 Gliwice, Poland; 3 Medical Laboratory CSD, Vasylkivska Street, 45, 02000 Kyiv, Ukraine; o.sulaieva@csd.com.ua; 4 Institute of Atomic Physics and Spectroscopy, University of Latvia, Jelgavas iela 3, LV-1004 Riga, Latvia; 5 Institute of Anatomy, Medical Faculty, University of Duisburg-Essen, 45147 Essen, Germany

**Keywords:** Mg-based implant, surface coating, plasma electrolytic oxidation, in vivo experiment

## Abstract

Biodegradable Magnesium (Mg) implants are promising alternatives to permanent metallic prosthesis. To improve the biocompatibility and with the aim of degradation control, we provided Plasma Electrolytic Oxidation (PEO) of pure Mg implant in silicate-based solution with NaOH (S1 250 V) and Ca(OH)_2_ (S2 300 V). Despite the well-structured surface, S1 250 V implants induced enormous innate immunity reaction with the prevalence of neutrophils (MPO+) and M1-macrophages (CD68+), causing secondary alteration and massive necrosis in the peri-implant area in a week. This reaction was also accompanied by systemic changes in visceral organs affecting animals’ survival after seven days of the experiment. In contrast, S2 300 V implantation was associated with focal lymphohistiocytic infiltration and granulation tissue formation, defining a more favorable outcome. This reaction was associated with the prevalence of M2-macrophages (CD163+) and high density of αSMA+ myofibroblasts, implying a resolution of inflammation and effective tissue repair at the site of the implantation. At 30 days, no remnants of S2 300 V implants were found, suggesting complete resorption with minor histological changes in peri-implant tissues. In conclusion, Ca(OH)_2_-contained silicate-based solution allows generating biocompatible coating reducing toxicity and immunogenicity with appropriate degradation properties that make it a promising candidate for medical applications.

## 1. Introduction

Biodegradable magnesium implants have emerged as a promising alternative to traditional permanent metallic implants in various medical applications [1]. Magnesium is an essential mineral in the human body and has been found to promote bone growth and healing (67% of the body’s Mg is deposited in bone tissue and serves as a dynamic store for the maintenance of Mg homeostasis) [2]. As the implant gradually degrades over time, it reduces the risk of long-term complications associated with permanent implants, such as stress shielding and implant-related infections. Over the years, significant research and advancements have been made in the field of biodegradable magnesium implants, enhancing their biocompatibility and mechanical properties for better clinical outcomes [3,4]. The biodegradable nature of magnesium implants allows them to gradually degrade over time, matching the healing rate of surrounding tissues. As the implant degrades, it transfers the load to the healing bone, promoting natural bone remodeling and regeneration [5,6]. Magnesium is the second most abundant, intracellular, divalent cation, and its corrosion products exhibit high biocompatibility. Moreover, unlike permanent implants, which can cause stress shielding and bone resorption, biodegradable magnesium implants alleviate these issues by gradually transferring the load to the bone as they degrade. Additionally, magnesium is relatively transparent to X-rays, making it easier for clinicians to monitor bone healing and implant degradation using standard radiographic techniques [2]. Finally, Mg-based implants do not require removal surgery, which significantly reduces surgery-associated risks [3].

Despite the promising properties, the application of Mg-based implants is limited due to possible rapid corrosion, especially in acidic conditions, which may lead to premature implant failure or gas formation. Since the beginning of Mg implant investigation (early 1990th), these complications, accompanied by granular tissue formation around implants, have limited wide application. Some researchers noted the limitations of Mg screw or plate application in orthopedic practice due to early corrosion. Controlling the degradation rate of magnesium implants is crucial to match the tissue healing process, and achieving a predictable degradation profile can be challenging. There are two substantial strategies to overcome these limitations: (i) the development of novel Mg-based alloys and (ii) the formation of protective coatings that control the degradation rate [4,7,8,9]. Application of rare earth elements (such as yttrium and gadolinium), zirconium, manganese, zinc, calcium, lithium, and strontium, can significantly increase mechanical properties and decrease the degradation rate of new Mg alloys. The main limitation of in vivo and clinical applications of such alloys is the increasing toxicity that accompanies Mg degradation [10]. Surface modification represents a highly effective approach, not only in managing the degradation behavior of magnesium (Mg) but also in enhancing its surface biocompatibility. Unlike altering the bulk structure and composition, surface modification offers a simpler and more convenient means to tailor the surface corrosion resistance of Mg alloys while retaining their advantageous bulk properties. Recent studies have demonstrated the remarkable potential of surface modification in significantly enhancing the corrosion performance of Mg alloy implants [11,12]. This treatment process creates a protective barrier, effectively shielding the implant from the surrounding body environment and promoting prolonged implant stability. By optimizing the surface properties, surface modification opens up new avenues for designing advanced Mg-based implants with improved biocompatibility and extended functional life. 

Our previous study demonstrated the ability to provide a protective coating for Mg alloys with the application of Plasma Electrolytic Oxidation (PEO) technique [13,14,15]. PEO coating technique can provide a stable ceramic layer with high biocompatibility on different metal alloys (Ti, Nb, Mg). Additionally, PEO is suitable for the modification of implants with irregular geometry that allow using it with different implant systems. Among different strategies, the application of a silicate-based solution of Ca(OH)_2_ demonstrated significant advantages in corrosion control and in-vitro cell response. We demonstrated appropriate cell adhesion and proliferation with no adverse effects that allow initiating in vivo experiments. An animal model is essential to prove the safety and degradation behavior of Mg implants in relevant media. In vivo study provides information about the influence of degradation products on surrounding tissues as well as general toxicity in case of product adsorption in the bloodstream. There are different models, including bone and subcutaneous implantation, that allow investigating host response to implanted Mg. In the current study, we choose the subcutaneous implantation model with the aim of fast assessment of peri-implant tissue reaction as well as liver and kidney structure at different time points after implantation of Mg implants with two different coatings.

## 2. Materials and Methods

### 2.1. Materials

The pure Mg (99.99%) used in this study was purchased from Polmag (Kedzierzyn-Kozle, Poland). Na_2_SiO_3_, NH_4_F, NaOH, and Ca(OH)_2_ were obtained from Sigma–Aldrich (St. Louis, MO, USA). To prepare the Mg cube samples with a 10 × 10 mm face size, they were ground using abrasive paper Hermes BW114 (Hermes Schleifmittel GmbH, Hamburg, Germany) with granulations of 400, 1000, 1200, and 1500, successively. Subsequently, the samples underwent ultrasonic cleaning in 2-propanol for 5 min. Prior to the Plasma Electrolytic Oxidation (PEO) treatment, the samples were rinsed with distilled water.

### 2.2. Plasma Electrolytic Oxidation (PEO)

The PEO process was conducted using an impulse current up to a fixed voltage with the aid of a high-voltage power supply (PWR 800H, Kikusui, Yokohama, Japan). The electrolyte solution (Table 1) (200 mL) utilized in the process was maintained at 15 °C and continuously stirred with a magnetic stirrer, while maintaining a constant current density of j = 100 mA/cm^2^. Following the PEO treatment, the samples were thoroughly rinsed with distilled water and left to dry in ambient air.

### 2.3. Surface Analysis

The surface morphology and elemental composition of the coatings were examined using scanning electron microscopy paired with an energy-dispersive X-ray spectrometer (SEM—JEOL JSM-7600F, JEOL Ltd., Tokyo, Japan; EDX—Edax Inc., Mahwah NJ, USA). The Trial Version Image-Pro 10.0.7 software was employed to describe the pore size and pore distribution.

Surface roughness was assessed using the tactile stylus method (Surftest SJ-301, Mitutoyo, Kawasaki, Kanagawa, Japan). The arithmetic mean of the sum of roughness profile values (Ra) and mean peak-to-valley height (Rz) were measured in triplicate.

The wettability properties of the coatings were characterized using a video-based optical contact angle-measuring instrument (OCA 15 EC, Series GM-10-473 V-5.0, Data Physics, Filderstadt, Germany). Optical analysis of ultra-pure water drops, about 0.5 µL in volume, placed on a solid surface was performed in five different positions, and the average value was recorded.

### 2.4. Animals and Surgery

The experimental protocol received approval from the Ethics Committee of Sumy State University (protocol number: 12; approval date: 11 December 2021). The study adhered to the guidelines outlined in the Handbook for the Care and Use of Laboratory Animals (1996) and Directive 2010/63/EU of the European Parliament and Council on the protection of animals used for scientific purposes (2010).

A total of sixty 6-month-old male white laboratory rats, with an average weight of 250–350 g, were selected for the study. All animals were kept under identical conditions, maintaining a constant temperature of 22 °C and a standard light-dark cycle, with unrestricted access to food and water throughout the research.

For the in vivo experiment, the animals were divided into three groups, each consisting of 20 animals, based on the type of samples administered. Two experimental groups received modified Mg samples using PEO in solutions S1 (250 V) and S2 (300 V), while the control group was given unmodified Mg samples. Animals were removed from the experiment on the 7th and 30th day of the study.

Anesthesia was administered via intramuscular injection, using 7 mg/kg of ketamine (AT “Farmak”, Kyiv, Ukraine) and 10 mg/kg of xylazine (Alfasan International B.V., The Netherlands). After aseptic procedures, a 1 cm incision was made in the shaved interscapular region. The implant was then placed in the subcutaneous adipose tissue with the same depth in each animal, and the wound was closed using two layers of absorbable sutures Vicryl 4/0, Ethicon (AgnThos, Lidingö, Sweden).

### 2.5. Histology

The animals were humanely euthanized after one week and one month post-surgery by administering an overdose of ketamine (100 mg/kg). Tissue samples from the implantation site, liver, and kidneys were fixed in a 10% neutral buffered formalin solution (pH 7.2) for 24 h. For histological analysis, 5 μm thick sections were prepared from paraffin-embedded tissue blocks and mounted on SuperFrost microscopic slides (Thermo Fisher Scientific; Waltham, MA, USA). The slides underwent standard deparaffinization using xylene and rehydration with a series of decreasing ethanol concentrations. Subsequently, the samples were stained with hematoxylin and eosin using conventional methods to enable visual evaluation of the histological specimens. All examinations were performed using a Carl Zeiss Primo Star microscope equipped with a Zeiss AxioCam ERc 5s digital camera and ZEN 2 (blue edition) software (Oberkochen, Germany). Morphometric parameters were assessed in 10 fields of view at ×100 magnification by two independent observers.

### 2.6. Immunohistochemistry 

To investigate the cellular mechanisms driving the inflammatory response triggered by Mg implants, immunohistochemistry was employed to visualize specific immune cell types involved in inflammation resolution. This included identifying polymorphonuclear leukocytes (PMNs) through MPO+ staining and distinguishing M1 and M2 macrophages through CD68+ and CD163+ staining, respectively. Additionally, the presence of myofibroblasts, crucial in granulation tissue, was assessed using alpha-smooth muscle actin (α-SMA) staining. Immunohistochemistry research was done in SCD Health Laboratory (Kyiv, Ukraine). 

Briefly, for immunohistochemistry (IHC), serial sections 4 µm thick were utilized. The tissues underwent deparaffinization and hydration. Endogenous peroxidase activity was blocked using 3% methanol in hydrogen peroxide. Antigen retrieval was carried out in a water bath at 98 °C using Tris EDTA or citrate buffer, followed by incubation with primary antibodies. Labeled polymer secondary antibodies (Envision Detection System, Agilent, Santa Clara, CA, USA) were added to the sections on the slides after washing. Peroxidase activity was detected using diaminobenzidine (DAB)—tetrahydrochloride liquid plus Chromogen System (Agilent) substrate, and the reaction was stopped with distilled water. Subsequently, the sections were counterstained with hematoxylin and mounted in Richard–Allan Scientific Mounting Medium (ThermoFisher, Waltham, MA, USA).

The numbers of MPO+, CD68+, CD163+, and α-SMA-positive cells were quantified per field of view at high magnification by two independent observers.

### 2.7. Statistical Analysis 

The findings were presented as the average value (M) and standard deviation (SD) for each sample or as a percentage increase compared to the control group. The normality of the data distribution was assessed using the Kolmogorov–Smirnov test. Group comparisons were performed using either analysis of variance (ANOVA) for normally distributed data or the Kruskal–Wallis test for non-normally distributed data. Statistical significance was determined at a significance level of *p* < 0.05 (less than 5%). All numerical data were processed using Graph Pad^®^ 8.0 software and Microsoft Excel 2016, running on the Windows 11 operating system.

## 3. Results and Discussion

### 3.1. Coatings Characterization

**Figure 1:**
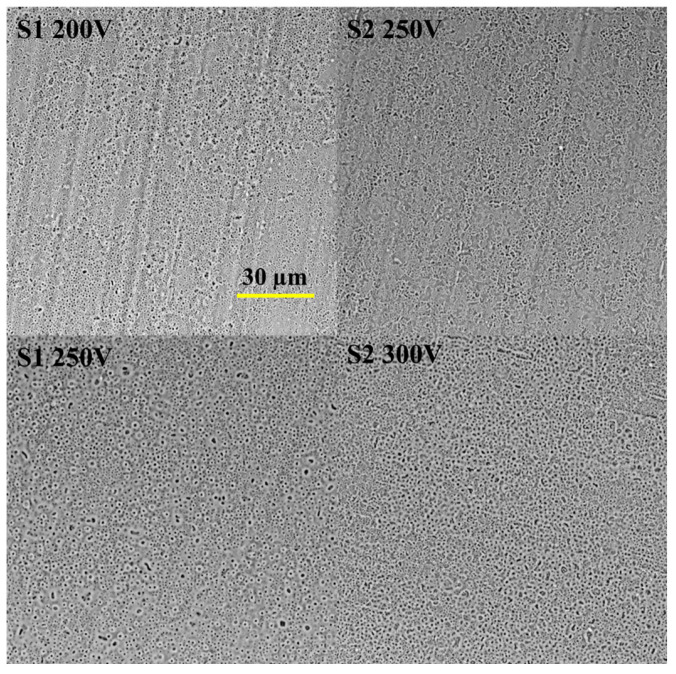
Scanning electron microscopy visualization of the Mg samples surface after the PEO process in different regimens.

Our previous investigation successfully achieved oxide-based coatings through the plasma electrolytic oxidation technique [16]. The surfaces of the samples exhibited a typical porous structure (Figure 1). Notably, the S1 electrolyte predominantly generated sub-micron pores with a size of up to 0.5 µm, while the S2 electrolyte resulted in larger pores with a mean diameter ranging from 1 to 5 µm. According to literature data, nanoporous surfaces are favorable substrates for protein adhesion, leading to subsequent cell attachment [17]. However, it is crucial to consider the chemical composition of the surface, as it can also influence cell adhesion [18]. The key chemical properties that affect cell adhesion include surface energy, surface charge, and bioactive factors. The silicate-based electrolyte ensured Si incorporation into the coating for both variants, with the S2 coating exhibiting a higher Si concentration. Conversely, the presence of Ca in the oxide coating was relatively low, and the anodizing voltage did not significantly impact the concentration. The stable oxide layer formed by the electrolytic process is responsible for the coating’s degradation resistance, biocompatibility, and clinical outcomes [19]. Notably, the S2 electrolyte enhanced the thickness of the oxide layer by up to seven times compared to the S1 coating. Additionally, the surface texture plays a significant role in the biocompatibility of the coating, particularly in relation to protein and cell adhesion. Our previous research revealed a high surface roughness value for the S2 coating. Both the roughness and functional groups on the surface can influence surface wettability, which, in turn, controls the adhesion of cellular proteins and affects cell behavior. Generally, cells tend to adhere and grow on surfaces that are moderately hydrophilic but not superhydrophilic (contact angle less than 5°) or superhydrophobic (contact angle greater than 150°) [20].

The obtained S1 surfaces displayed hydrophilic properties, with contact angle values of 26.22° and 22.7° for the 200 and 250 V samples, respectively, indicating their hydrophilic nature. On the other hand, the wettability of the S2 surface was comparatively lower, with a contact angle of 16.61°, attributable to the addition of Ca(OH)_2_. Furthermore, an increase in the applied voltage to 300 V led to a decline in the contact angle, approaching 0°, signifying enhanced hydrophilicity.

Based on our previous results [16], we selected two coatings for in vivo experiments: S1 250 V (NaOH-contained) and S2 300 V (Ca(OH)_2_-contained solution).

### 3.2. Histology Assessment

**Figure 2:**
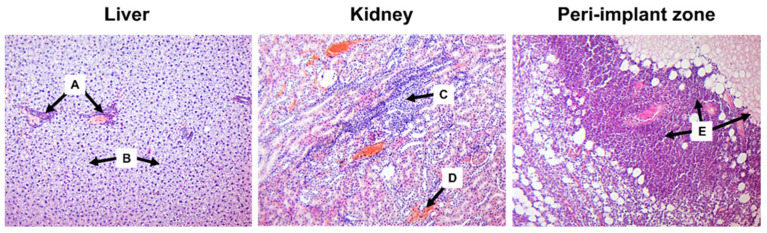
Tissue of the liver, kidneys, and implantation site of S1 250 V in rats after 7 days of observation. A—periportal inflammatory infiltration, B—hepatocytes with severe fatty degeneration, C—inflammatory infiltration of the renal parenchyma, D—hemorrhage, E—pronounced inflammatory and destructive-necrotic changes in the peri-implant zone. Hematoxylin and eosin staining. Magnification ×100.

Despite the well-structured surface and coating, S1 250 V implants provided significant local and systemic harmful effects. The majority of animals did not survive after the first week of observation. None of the rats survived until the second scheduled time point of the experiment (30 days of observation). After seven days of the experiment, they had numerous cavities and massive necrotic changes around the site of implantation, associated with the prominent inflammatory reaction, were found. Cavity wall was edematous and infiltrated by PMN (Figure 2).

The magnitude of the inflammatory reaction was associated with the prevalence of PMN infiltration correlating with the number of MPO-positive cells. Such response was accompanied by a prevalence of CD68+ macrophages, whose number overweighted scattered CD163+ cells (*p* < 0.001). These features could reflect the altered resolution of inflammation that was supported by poor granulation tissue formation and a low number of disorganized a-SMA-positive myofibroblasts. At the periphery of the peri-implant area, granulation tissues with dilated blood vessels and hemorrhages were found. However, it comprised a loose network of myofibroblasts intermitted with CD68+ cells and neutrophils. Thus, S1 250 V implants caused adjacent tissue damage and exaggerated inflammatory reaction with massive inflation by innate immunity cells and deficiency of tissue repair.

In addition to local injury, the profound systemic adverse effect of S1 250 V implants affecting liver and kidney structure was revealed. Implant-induced portal inflammation associated with hepatocellular fatty degeneration, swelling, areas of necrosis, and hemorrhage were found in the liver (Figure 2). In the kidneys, tubule-interstitial changes included tubular epithelium damage (with both necrosis and apoptosis features), extensive hemorrhages, and intense infiltration of the interstitial space by lymphocytes and macrophages.

**Figure 3:**
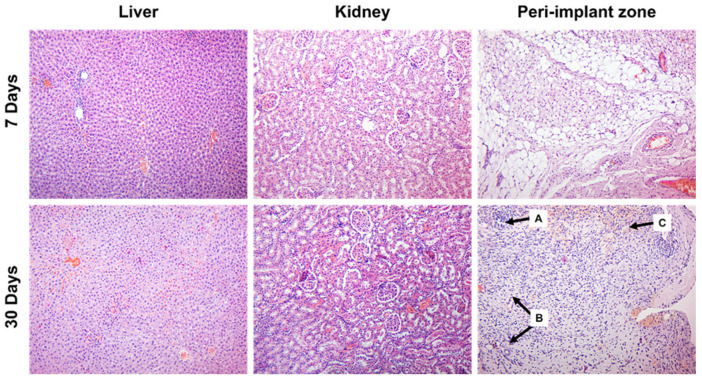
Liver and kidney tissue, as well as the implantation site of S2 300 V in rats in 7 and 30 days of observation. A—focal inflammatory infiltration, B—granulation tissue, C—hemorrhage. Hematoxylin and eosin staining. Magnification ×100.

The outcome of the animals which were implanted with S2 300 V was beneficial. All the animals survived, so the assessment of per-implant tissues and visceral organs was possible both in the 7th and 30th days of the experiment. The local effect of S2 300 V implants was less harmful. Although the peri-implant zone also possessed inflammatory infiltration on the 7th day of the experiment, its intensity was significantly less prominent (Figure 3). 

Inflammatory infiltration included fewer MPO+ neutrophils (*p* < 0.001) and CD68+ (*p* = 0.017) cells, while CD163+ macrophages were more numerous (*p* < 0.001) as compared to the S1 250 V group. At the same time, forming granulation tissue was rich in narrow vessels and myofibroblasts whose density was significantly higher than in the S1 250 V group (*p* < 0.001). 

After 30 days of the experiment, no remnants of the implants were found in the examined material. Peri-implantation area comprised the connective tissues with remnants of maturing granulation tissue, and focal lymphohistiocytic infiltration was observed (refer to Figure 3, lower row). 

In contrast to the S1 250 V group, the use of S2 300 V implants was not associated with a harmful systemic effect. Remarkably, the subcutaneous implantation of S2 300 V did not induce any apparent pathological changes in the internal organs (see Figure 3). The morphological features of the liver and kidney tissues in the experimental rats closely resembled those of the normal state. Only in some biopsy samples, mild focal lymphocytic infiltration in the portal tracts and mild swelling were observed on the 30th day of observation.

Histological examination of biopsy samples retrieved from the implantation sites of rats with the S2 300 V implant revealed noteworthy findings after seven days of observation. Specifically, focal lymphohistiocytic infiltration was observed within the fibro-fatty tissue, accompanied by localized areas of granulation tissue growth and small perifocal hemorrhages (see Figure 3, upper row). These observed alterations are likely attributed to the traumatic injury inflicted on the soft tissues during the subcutaneous implantation of the S2 300 V device.

On the 30th day of the experiment, no remnants of the implants were found, demonstrating the complete degradation of the implanted biomaterials. The tissues at the site of intervention demonstrated restoration of the histological structure with scarce immune cells and isolated foci of granulation tissue (Figure 3, lower row).

To evaluate the potential overall toxic effects resulting from the resorption of the implanted material S2 300 V, we conducted separate examinations of liver and kidney tissues in rats after 7 and 30 days of the experiment. Remarkably, the subcutaneous implantation of S2 300 V did not induce any apparent pathological changes in the internal organs (see Figure 3). The morphological features of the liver and kidney tissues in the experimental rats closely resembled those of the normal state. Only in some biopsy samples, mild focal lymphocytic infiltration in the portal tracts and mild swelling were observed on the 30th day of observation.

The absence of local and systemic proinflammatory and toxic effects of the implanted S2 300 V in rats was consistently observed, both after one week (partial resorption) and after one month of observation (complete resorption of the implant).

In stark contrast, the outcomes were entirely different in animals subcutaneously implanted with S1 250 V. It is crucial to highlight that the majority of animals did not survive beyond the first week of observation. None of the rats survived until the second scheduled time point of the experiment (30 days of observation). When examining the tissues of the implantation site after seven days of the experiment, we observed pronounced inflammatory and destructive-necrotic changes in the areas adjacent to the implant. The closer regions were characterized by necrotic tissue, which was surrounded by granulation tissue and delicate maturing connective tissue at the periphery. The adjacent fibrous-adipose tissue showed intense mixed-cellular inflammatory infiltration, consisting of both granulocytes and agranulocytic leukocytes (lymphocytes and macrophages) (Figure 2). Residual connective tissue and foci of hemorrhage were visualized within the inflammatory infiltrate and necrotic areas.

The toxic impact of the S1 250 V implant was not limited to the tissues adjacent to the implantation site, rather, it extended to other organs, leading to significant pathological changes. Upon examining the liver and kidney tissues after seven days of the experiment, we observed remarkable alterations indicative of toxic hepatitis and toxic tubulointerstitial nephritis, respectively. The thick coating in S1 250 V could provide fast degradation and release of Mg and its salts that provide toxic effects. 

In the liver tissue, clear signs of toxic hepatitis were evident, characterized by prominent periportal inflammatory lymphohistiocytic infiltration, hepatocellular fatty degeneration, swelling, areas of necrosis, and hemorrhage (refer to Figure 2).

Similarly, the kidneys exhibited comparable changes. During this seven-day period, the renal parenchyma displayed indications of toxic tubulointerstitial nephritis. Among the relatively intact glomeruli, renal tubules with dystrophic changes in the nephroepithelium (mainly proteinaceous degeneration) were identified, some of which were necrotic or in an apoptotic state. The renal interstitium showed intense infiltration of leukocytes, predominantly lymphohistiocytic in nature. Additionally, it appeared swollen and displayed extensive hemorrhage. Furthermore, stasis and foci of vasculitis were observed in both the liver and kidney vessels (see Figure 2).

The more favorable tissue responses to S2 300 V sample could be related to the coating thickness. The thicker oxide layer could provide better prevention against Mg ion release, which prevents the formation of an alkaline toxic environment around the implant [21]. Similarly, other parameters specific to the implant, such as the geometry, the chemical composition of the material(s), wettability, and the surface roughness, also influence the biocompatibility properties of the material. As noted by Carlos Nelson Elias, one possible method of improving implant biocompatibility is to increase surface roughness and decrease the contact angle [22]. S2 300 V type of coating showed both characteristics of high surface development and super hydrophilic properties.

### 3.3. Immunohistochemical Analysis

Both groups exhibited an inflammatory response around the site of Mg-implant application, but there were significant differences in the severity of the reaction.

**Figure 4:**
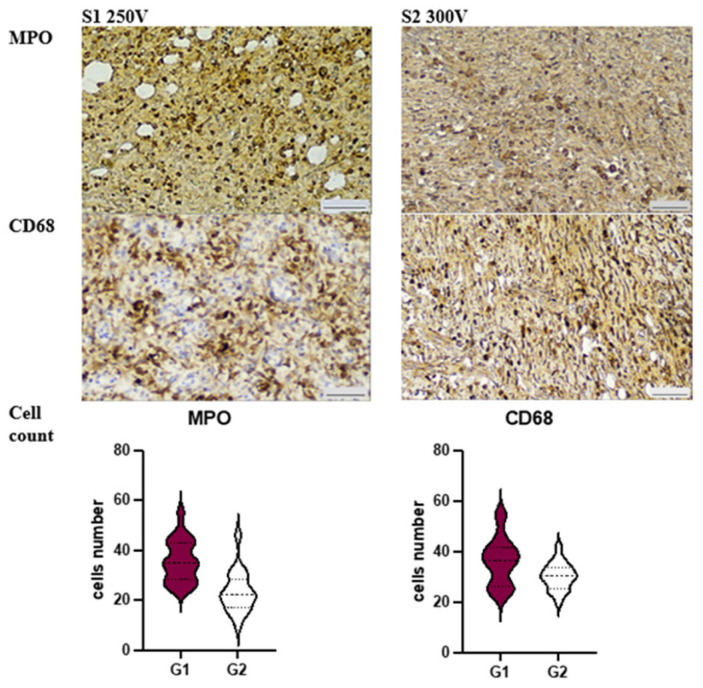
Characteristics of immune cells contributing to acute inflammatory reaction under Mg-implants application. Immunohistochemistry.

**Figure 5:**
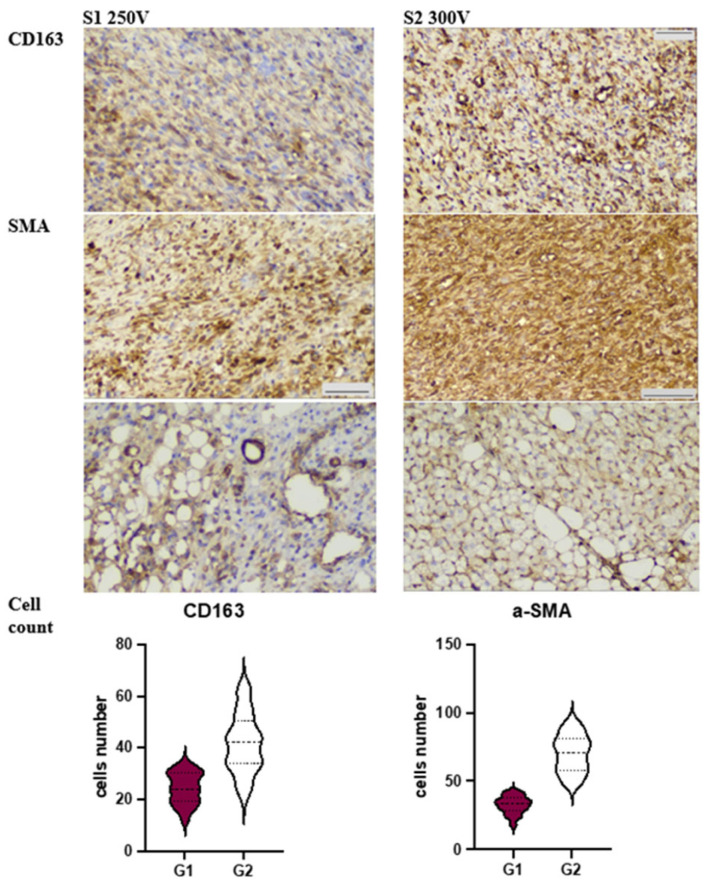
Characteristics of cells contributing to tissue repair under Mg-implants application.

In S1 250 V, we observed a prevalence of PMN (polymorphonuclear leukocyte) infiltration, which correlated with the number of MPO (myeloperoxidase)-positive cells (see Figure 4). This response was accompanied by an abundance of CD68+ macrophages, outnumbering the scattered CD163+ cells (*p* < 0.001). These features suggest an altered resolution of inflammation, which was further supported by poor granulation tissue formation and a low number of disorganized a-SMA (alpha-smooth muscle actin)-positive myofibroblasts (refer to Figure 5).

On the other hand, the soft tissue reaction in S2 300 V revealed significantly lower numbers of MPO+ cells (*p* < 0.001) and CD68+ macrophages (*p* = 0.017), while CD163+ macrophages were more abundant (*p* < 0.001). Interestingly, the dermis demonstrated an enormous density of aSMA+ cells, which were significantly higher in number compared to Group 1 (*p* < 0.001). Additionally, we identified numerous aSMA-positive cells, which aligns with the well-known fact that pericytes of microvessels contribute as a source of myofibroblast lineage under repair conditions. Application of modified Mg-implants S2 300 V was associated with reduced MPO+ and proinflammatory CD68+ cell count.

Application of modified Mg-implants S2 300 V was associated with M2-macrophages polarization with high aSMA+ cells count in dermis and hypodermis, which underwent browning.

The lower thickness of the S1 250 V coating could cause rapid degradation of Mg implants and amplifies the foreign body response by promoting initial inflammation. The results reveal that the initial local release of degradation products from Mg implants triggers a robust proinflammatory response in soft tissue with a toxic effect on rat organisms [23].

The application of Mg-based implants is usually accompanied by inflammation of the peri-implant tissues together with the gaseous cavities resulting from the gaseous accumulation generated by the degradation of the Mg implant [24,25]. According to our data, application of Mg-based implants with S1 coating was associated with gaseous void formation and profound inflammatory reaction, associated with numerous PMNL and M1-macrophages recruiting. Previously it was indicated that the release of Mg degradation products can initiate chemotaxis of leukocytes upregulating proinflammatory activation. In Ben Amara H study, authors showed that Mg-based implants induced expression of inducible nitric oxide synthase and toll-like receptor-4 expression, whose levels rose up to the sixth day of the experiment [23]. These data correlate with our findings in the S1 250 V group demonstrating intense reaction of innate immunity cells on the seventh day after implantation. Notably, the major type of inflammatory cells were neutrophils exhibiting MPO activity and M1-macrophages demonstrating proinflammatory activity. Beyond the role as a first-line cell of innate immunity responding to damage and foreign materials, prolonged or exaggerated recruitment of PMNL and M10 macrophages results in extensive secondary damage associated with proinflammatory cytokine production (including IL-1b, IL-8, IL-33) and active oxygen species generation with further exudation and secondary tissue alteration [25].

On the other hand, different compositions of Mg alloys and their combination with various coatings can affect cell viability, differentiation, and functioning [26]. As it was mentioned above, our previous research revealed a high surface roughness value for the S2 coating, impacting surface wettability and interplay with adhesive molecules, modifying cells’ reaction, and behavior. According to the obtained data, S2 coating provides beneficial effects on Mg degradation and implant-host interactions.

Interestingly, the impact of Mg-containing implants on vascularization associated with upregulated VEGF expression was reported [27]. One of the key sources of VEGF is M2 macrophages exhibiting anti-inflammatory and pro-regenerative potential. According to the obtained in our study data, coating with Ca(OH) shifts the surrounding tissues’ response to Mg-containing implant towards M2-macrophages polarization. As a result, the resolution of inflammatory reaction and shift to effective tissue repair at the site of implantation was observed.

## 4. Conclusions

In this study, we investigated the in vivo behavior of two surface-modified biodegradable magnesium implants, S1 250 V and S2 300 V, using a rat subcutaneous implantation model. The results demonstrated that the S2 300 V implant exhibited more favorable tissue responses compared to S1 250 V. S2 300 V showed reduced inflammation, enhanced biocompatibility, and complete resorption after 30 days, suggesting its potential as a promising candidate for medical applications.

The pronounced inflammatory and necrotic changes observed with S1 250 V implants, along with the lack of animal survival beyond the first week, highlight the importance of careful material selection and surface modification to ensure implant success.

On the other hand, the well-tolerated tissue response and complete degradation observed with S2 300 V implants underscore the significance of surface modification in tailoring the degradation rate and enhancing the overall biocompatibility of magnesium implants.

Furthermore, the immunohistochemical analysis revealed distinct cellular responses between the two groups, with S1 250 V showing M1 macrophages and S2 300 V displaying M2 macrophages and αSMA+ myofibroblasts. These findings suggest that S2 300 V surface modification promotes a more favorable tissue healing environment, fostering a pro-regenerative response compared to the proinflammatory environment observed with S1 250 V.

Overall, the success of surface-modified S2 300 V implants in reducing inflammation, improving biocompatibility, and enabling complete resorption highlights the importance of tailored surface modifications for enhancing the performance of biodegradable magnesium implants. These results contribute valuable insights towards developing advanced magnesium-based implant materials with improved clinical outcomes and reduced long-term complications. Further research is warranted to explore the full potential of surface-modified magnesium implants and to optimize their properties for a wide range of medical applications.

The further in-depth discovery of the relationship between Mg-alloys with regard to coating and host response, including both local spatiotemporal changes and systemic effects, is essential for tailoring Mg-implants for clinical application.

## Figures and Tables

**Table 1:** The chemical composition of the electrolyte baths and the anodizing parameters for the PEO process.

Electrolyte Type	Concentration of Electrolyte Components; g·dm^−3^				Anodizing Voltage
	Na_2_SiO_3_	NaF	NaOH	Ca(OH)_2_	
S1	10	5	10		200, 250
S2	10	5		10	250, 300

## Data Availability

The data is available on request in corresponding authors.
